# Response to letter regarding “Alloimmunization in dogs after transfusion: A serial cross‐match study”

**DOI:** 10.1111/jvim.16628

**Published:** 2023-01-13

**Authors:** Lisa Herter, Christiane Weingart, Nina Merten, Nicole Bock, Roswitha Merle, Barbara Kohn

**Affiliations:** ^1^ Faculty of Veterinary Medicine Clinic for Small Animals, Freie Universität Berlin Berlin Germany; ^2^ Department of Veterinary Medicine Institute for Veterinary Epidemiology and Biostatistics, Freie Universität Berlin Berlin Germany

Dear Editors,

Thank you for giving us the opportunity to respond to the letter of Prof Dr Urs Giger in which it is questioned whether the positive cross‐match results indicate alloimmunization or just unspecific agglutination reactions, and whether the applied tube method was appropriate.

Alloimmunization can be detected by screening for specific antibodies or by cross‐matching with the original donor.^1^ Different cross‐matching procedures are available. The conventional tube method has been used in several canine and feline studies to detect alloantibodies.^2^, ^3^ In addition to the conventional tube testing other methods including gel tube, and immunochromatographic strip methods are available for cross‐matching. Cross‐matching can be enhanced by adding antiglobulins. Although some authors discussed whether the tube method might be too sensitive and detect clinically irrelevant alloantibodies,^4^ the tube method is still considered the gold standard in veterinary medicine.^1^ However, we fully agree that it is difficult to conclusively document RBC alloimmunization, that is to determine which red blood cell (RBC) antigen is targeted by antibodies, and to exclude nonspecific agglutination reactions.

A further question was if the addition of antiglobulins is needed to differentiate between antibody detection and unspecific agglutination reactions. Comparing different cross‐matching protocols, the addition of antiglobulins while performing the tube technique enabled an increase in sensitivity.^4^ Therefore, by adding antiglobulins in our study, lower titers of antibodies might have been detected. In a recent study evaluating different cross‐matching methods in comparison to a conventional tube method, the antiglobulin‐enhanced gel tube method showed concordant results in all (10/10) compatible cross‐match tests, in 6 of 10 tests with a mild microscopic agglutination, and in all (20/20) tests with a macroscopic agglutination.^5^ In human medicine the different cross‐matching techniques seem to be of varying sensitivity detecting different alloantibodies. It is assumed that no method will detect all antibodies of clinical relevance. Comparable data are not available in veterinary medicine.

In human medicine extended blood typing as well as antibody screening in addition to cross‐matching has been used before a transfusion to reduce the rate of alloimmunization. In dogs more than 12 blood groups are known and it is assumed that there are other as yet undiscovered blood groups. In veterinary medicine extended blood typing as well as antibody screening is difficult to perform in a clinical setting because of a limited supply of test reagents. Cross‐matching is therefore a possibility to identify incompatibilities against other blood groups as well as to detect irregular alloantibodies. Extended blood typing was not performed in this study but should be considered in further studies.

As in our study, alloimmunization has been shown in several species within 4 days after transfusion. In human medicine, although alloimmunization was mostly detected at a later date after transfusion, antibodies were found in one study within 2 to 3 days in 2.3% of all immunized patients.^6^ In cats alloimmunization was detected within 2 days after transfusion by performing serial cross‐matching.^2^ The first alloimmunization in dogs was described against the Dal‐antigen in one study on day 4 after the transfusion.^7^ In human medicine it was suggested that ideally, RBC antibody tests should be performed twice, the first time shortly after transfusion, to detect boosting of existing antibodies or fast appearing new antibodies and a second time to detect slower developing antibodies.^6^

In our study, all cross‐match tests were negative before transfusion. In studies of dogs performed recently naturally occurring alloantibodies could be detected in transfusion naïve dogs.^3^ Although these alloantibodies are believed to be of no clinical relevance, one study demonstrated that dogs that underwent cross‐matching before the transfusion had a significantly greater mean increase in hematocrit after transfusion than dogs that did not undergo cross‐matching.^3^ It is supposed that a transfusion of noncompatible blood could lead to an increase in alloantibodies and to a shortened life span of the transfused erythrocytes or possibly lead to complications with further transfusions. Therefore, some authors even suggest to include cross‐matching in routine pretransfusion testing.^3^ A practice which we do not perform in our hospital in dogs. Even with a compatible cross‐match test, appropriate RBC survival, complete elimination of transfusion reactions, or both are not guaranteed as low titers of anti‐RBC antibodies might not cause enough agglutination for detection, but could still result in a transfusion reaction.^1^ These antibodies could also lead to a rapid anamnestic response and early alloimmunization after transfusion.

However, as we discussed in our article the clinical relevance of weak agglutination reactions is unclear. Recipients with very low levels of alloantibodies might not mount an immediate response to a transfusion, but will upregulate alloantibody production after transfusion.^1^ In a clinical setting delayed transfusion reactions are difficult to prove, for example, because of underlying diseases of the recipient, and therefore might be unnoticed.

It was questioned if the agglutination reaction detected in our study was too mild and resolved too quickly in some dogs and therefore might not be because of alloimmunization. Although alloantibodies built against dog erythrocyte antigen (DEA) 1 and Dal antibodies have a certain longevity, it is unclear how long other antibodies will persist. In human medicine, the evanescence of blood group antibodies is a known risk with 24% of evanescent antibodies detectable for less than a month and 5.6% for less than a week.^8^ Further studies are needed to investigate how different alloantibodies behave after a transfusion in dogs.

As already mentioned, the results of the tube procedure can be influenced by various factors. Rouleaux formation or inadequate RBC washing can cause false positive results, antibodies can be eluded during the washing phases, and formed agglutinates can break up when tapping against the tube for resuspension. Furthermore, the evaluation of mild microscopic positive cross‐match results can be difficult. Therefore, the experience of the person performing the cross‐match is of particular importance. In our study, cross‐matching was only performed by trained personnel. Rouleaux formation was excluded and the result was reevaluated by an experienced laboratory technician, blinded to the results of the first examiner.

Furthermore, the storage duration of the donor samples, an inferior quality of transfused RBCs, and inflammatory responses in the recipient might provoke false positive cross‐match results. As discussed in our article, various factors, like storage duration, transfused blood volume, and the underlying disease of the recipient were examined for their influence on the cross‐matching result. Although, there was no significant association between those factors and the appearance of a positive cross‐match test result within 4 days after transfusion, a potential influence cannot be ruled out. Underlying inflammatory diseases might have an influence on nonspecific agglutination reactions but also on the alloimmunization rate.

In practice, several factors must be considered when deciding whether to transfuse a dog including the availability of blood products (exclusion of blood products with a low level of cross‐match incompatibility can limit blood product availability), the time needed for compatibility testing, and the clinical condition of the recipient. The decision for cross‐matching in a clinical case needs to be balanced with the urgency of the dog's clinical state since cross‐matching all recipients and donors might not be practically or financially possible. Even if our findings are not of clinical concern in most of the dogs, we think that our findings are of scientific interest and should give rise to further studies.
